# A Multiscale Mechanistic Framework for Polydopamine Dyeing in Ultrablack Wool Textiles

**DOI:** 10.1002/advs.77312

**Published:** 2026-08-24

**Authors:** Hansadi Jayamaha, Kumar Siddharth, Kyuin Park, Mia Bressler, Larissa M. Shepherd

**Affiliations:** ^1^ Department of Human Centered Design College of Human Ecology Cornell University Ithaca New York USA; ^2^ School of Civil and Environmental Engineering Cornell University Ithaca New York USA

**Keywords:** dopamine polymerization, photonic textiles, polydopamine dyeing, ultrablack textiles, wool hierarchy

## Abstract

Polydopamine (PDA)‐dyed wool textiles offer a potentially scalable route to ultrablack materials that combine wide‐angle reflectance suppression, flexibility, breathability, and biocompatibility. Their development, however, has remained largely empirical, lacking structural and mechanistic foundations for PDA dyeing, and prior ultrablack wool systems have been restricted to merino‐based single‐jersey fabrics. Here, we establish a multimodal structural and mechanistic framework for PDA dyeing by integrating dopamine polymerization chemistry, fiber surface chemistry and ultrastructure, as well as macroscopic fabric architecture. These factors collectively govern dye uptake and optical response. Dye formulation optimization reduces the dyeing time to 15–30 min across merino and generic wool fibers spanning diverse fabric architectures. Notably, fabric architecture emerges as a dominant design variable for controlling wide‐angle optical response, a previously underappreciated lever for engineering photonic textiles. Mechanistic analysis of dopamine polymerization reveals PDA formation in wool as an absorption‐driven dyeing process rather than a purely surface‐coating process, whose uptake and interactions within the porous fibrous matrix contribute to near‐zero reflectance when plasma‐treated. Together, these findings provide a material design blueprint for scalable ultrablack wool textiles and offer broader principles for flexible photonic devices.

## Graphical abstract

Ultrablack photonic textiles are enabled through polydopamine (PDA) dyeing, which is governed by the interplay of PDA chemistry, wool surface topology and chemistry, and hierarchical fiber–yarn–fabric architecture. Fabric architecture emerges as a primary and previously underappreciated design lever for wide‐angle optical performance. Together, these findings offer a transferable blueprint for engineering photonic textiles.

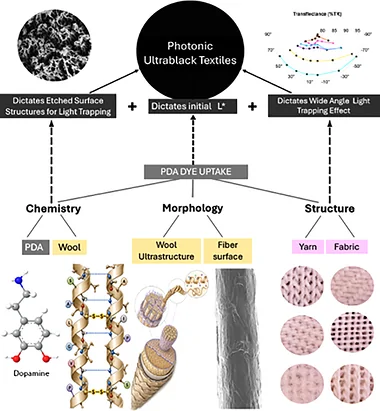

## 1 Introduction

Nature often serves as inspiration for the development of functional materials. One such example is mussel adhesive proteins, which are a class of biomolecules that show wet adhesion to a wide range of inorganic and organic surfaces [1, 2, 3, 4, 5, 6]. These proteins possess two key features: (1) a high content of catechol (3,4‐dihydroxybenzene) groups arising from 3,4‐dihydroxy‐L‐phenylalanine (L‐DOPA), and (2) a high density of primary and secondary amines from lysine and histidine residues [7]. Studies on the polymerization of dopamine (DA), an analog of L‐DOPA, date back to the 1970s [8, 9], and the structural similarity of polydopamine (PDA) to eumelanin, a pigment found in numerous animal species, has been well reported [10, 11, 12, 13, 14, 15]. More recently, Lee et al. demonstrated that DA can polymerize to form thin PDA films with surface adhesion properties resembling those of mussel proteins [16]. PDA's simple one‐step process, substrate‐independent adhesion, and melanin‐like photophysical properties enable its use in diverse fields [17, 18], including biomedical engineering [19, 20, 21], energy storage devices [22, 23, 24], environmental and catalytic technologies [25, 26], and photonics [19, 27].

While PDA micro‐ and nanoparticles have been embedded into diverse material platforms [19, 20, 28, 29], the predominant approach involves the use of PDA as a thin‐film or coating on a substrate's outer surface [17, 30, 31]. More recently, PDA has been used as a textile dye [14, 32]. DA polymerization with a two‐electron oxidant, sodium periodate (SP; NaIO_4_), penetrates the surface of merino wool fibers. In this system, PDA interacts with both the surface and internal fiber structure, creating a durable black coloration [14, 32]. This fiber‐dyeing behavior enables a particularly remarkable application: ultrablack textiles (i.e., materials that reflect less than 0.5% of visible light [32, 33, 34]). PDA dyed merino wool (PDAmW) exhibits monotonic absorbance in the visible to near‐infrared (NIR) region, analogous to eumelanin in natural ultrablack organisms [33, 35, 36, 37]. By plasma etching PDAmW, a new class of flexible and breathable ultrablack textile is attained, achieving a %R_avg_ ∼ 0.13 [32].

Wool fibers demonstrate significant genetic, proteomic, and structural variations based on the animal breed, maturity, as well as external factors such as nutrient stress, geographic location, and climate conditions [38, 39], which affect their physical and chemical fiber properties [38, 39, 40, 41, 42]. Dye uptake and cost are known to be associated with variations in fiber diameter, surface morphology, and internal structural assembly, with finer fiber grades such as merino wool being considerably more expensive than coarser and more widely available alternatives [38, 43]. To date, the darkest reported ultrablack wool textiles have been limited to merino single jersey fabrics [32].

In terms of mechanism, it is well established that PDA formation is initiated by DA oxidation under mildly alkaline conditions (pH ≥ 7.5) in the presence of dissolved oxygen, or by chemical oxidation using oxidants such as SP, ammonium peroxydisulfate ((NH_4_)_2_S_2_O_8_), potassium permanganate (KMnO_4_), copper sulfate (CuSO_4_), and Fe(III) salts [6, 9, 17, 44]. Oxidation is followed by intramolecular cyclization and isomerization, with covalent and noncovalent interactions serving as the driving forces for polymerization and self‐assembly. The sequence of cyclization, types of oligomers, and specific pathways to PDA formation and adhesion to surfaces, however, remain debatable in the literature and vary across different systems [18, 45, 46, 47]. Several studies examining PDA as a bridging layer also suggest that it binds to protein‐based substrates through either noncovalent interactions or covalent reactions with nucleophilic groups, including catechol‐thiol and catechol‐amine bonds formed by Michael‐type addition and/or Schiff base reactions [6, 44, 48]. The mechanism and chemistry underlying PDA‐wool dyeing, however, cannot be fully understood from existing literature, which is limited to surface‐level PDA adsorption or coatings.

The following knowledge gaps motivate the present study. First, although ultrablack wool has been demonstrated on single jersey merino wool, the influence of wool fiber type and fabric construction on PDA dye uptake and final optical performance remains insufficiently understood. This has limited the extension of the approach to more economical and readily available wool types and textile architectures. Second, PDA is widely described in the literature as a universal surface coating, whereas its behavior during textile dyeing, including absorption, penetration, and immobilization within keratin fibers, has not been characterized and may provide distinct insight into the PDA formation process. In this work, we address these gaps by investigating the molecular, fiber‐scale, and fabric scale contirbutions that enable PDA dyeing in wool textiles, focusing on how dopamine‐derived species are absorbed within the wool keratinous matrix, and how these processes couple with fabric architecture to enable broadband, wide‐angle optical response.

Therefore, we determine the role of PDA in fabricating ultrablack materials and optimize the dyeing parameters in terms of time, temperature, concentration, and solvent. The effect of surface chemistry, morphology, and ultrastructure of two distinct wool types on dye uptake is then characterized using ATR‐FTIR, SEM, TEM, HAADF‐STEM, and Raman spectroscopy. The effect of fabric construction on dye uptake as well as light‐material interactions (visible to NIR regime), which is critical for the ultrablack effect, are analyzed using UV–vis–NIR diffuse reflectance accessory (DRA) measurements. Finally, we propose a mechanistic pathway of PDA formation and wool dyeing via surface absorption of PDA intermediate species through liquid (in situ) and solid‐state NMR, XPS, and UV–vis spectroscopy. By providing a holistic picture of fiber‐to‐fabric structural contributions alongside new mechanistic insights into PDA dyeing, a framework is provided for engineering PDA‐functionalized fabric‐based optical devices with optimum wide‐angle photonic performance.

## 2 Results

### 2.1 Role of Polydopamine in Fabricating Ultrablack Textiles

**FIGURE 1:**
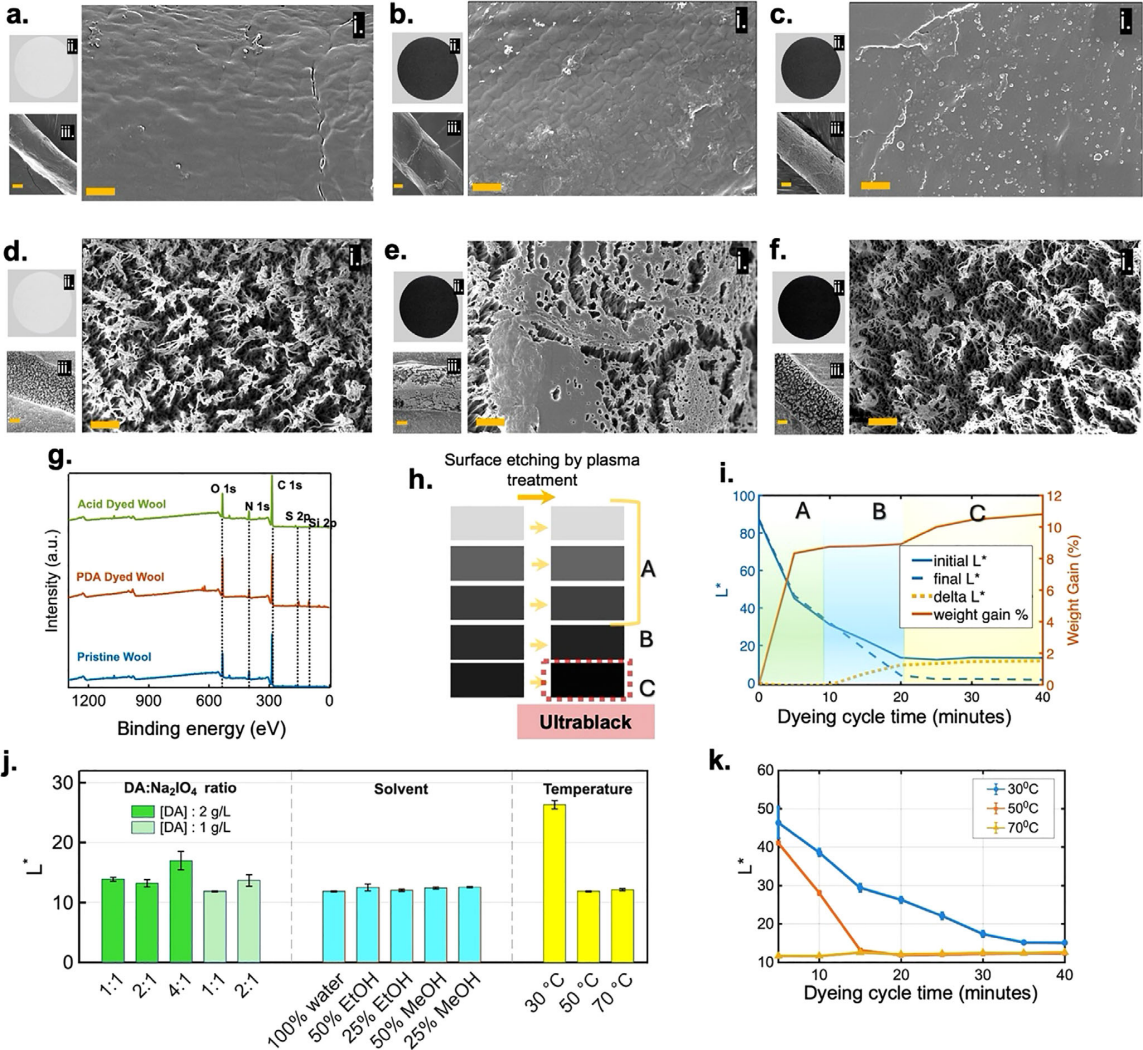
Role of polydopamine as a dye in fabricating ultrablack textiles: (a) pristine merino wool (mW), (b) polydopamine‐dyed merino wool (PDAmW), (c) acid black‐dyed merino wool (AmW), (d) plasma‐treated mW, (e) plasma‐treated PDAmW, and (f) plasma‐treated AmW. For (a–f): (i) higher‐magnification SEM images of fibers (scale bar is 1 micron), ii. photographs of the fabrics, and iii. lower‐magnification SEM images of fibers (scale bar is 4 microns). (g) Wide scan XPS data of W, PDAmW and AmW showing elemental composition. (h) Visual representation of the color change when shades of PDAmW fabrics are plasma etched for 80 min at 40 W power. (i) Effect of dyeing time on the PDA weight gain and L* values of PDAmW and plasma‐etched PDAmW. (j) Effect of dyeing parameters (concentration, solvent v/v%, and temperature) on the L* of the PDAmW. (k) Progression of L* with dyeing time at 30°C, 50°C, and 70°C.

PDA‐dyed merino wool (PDAmW) is susceptible to metal‐induced anisotropic plasma etching, creating a hierarchical surface structure with average total reflectance, %R_avg_ ∼ 0.13% [32]. In this study, we explain the role of PDA, the black pigment, and its chemistry in achieving this remarkable optical performance. We compare PDAmW with a commercially available acid‐dyed merino wool (AmW). The AmW is used as a reference material to highlight the distinct optical and surface chemical behavior of PDAmW rather than to establish a universal superiority of PDA over all acid dyes. Pristine merino wool (mW), PDAmW, and AmW are first plasma etched using previously established plasma etching conditions (80‐min treatment at 40 W power [32]). We observe anisotropic etching across the entire fiber surface for mW and PDAmW, whereas AmW shows an irregularly etched surface (Figure 1a‐fi, Figure S1). To understand this behavior, surface elemental composition is recorded using X‐ray photoelectron spectroscopy (XPS) and summarized in Table 1 and Table S1. All three samples show distinct C/O and C/N ratios, with trace amounts of silicon, likely from textile finishing, which may also affect etchability. Most notably, as C/O and C/N decrease, etchability increases in the order AW ≪ W < PDAW (Figure 1a–f). This is in agreement with a study by Moon et al., which explains the effect of composition on a material's etchability [49]. Table 1 and Figure S2 also contain spectra and data collected from the deconvoluted FTIR amide I band (1700 − 1600 cm^−1^) [42, 50], which corresponds to N─H bending and C─N stretching vibrations. Deconvolution enables estimation of the relative content of α‐helix and β‐sheet structures. Although studies suggest the α‐helix/β‐sheet ratio may contribute to long‐range order and higher crystallinity [41], another parameter that determines etchability [49], no direct correlation between the α‐helix/β‐sheet ratio and etchability is observed.

**TABLE 1:** Summary of chemical and structural data of the mW, PDAmW and AmW.

	mW	PDAmW	AmW
L* (initial)	93.72 ± 0.24	13.60 ± 0.40	12.32 ± 0.15
L* (final)	93.71 ± 0.18	0.69 ± 0.16	4.96 ± 0.23
C/O	4.98	2.53	7.57
C/N	9.09	7.75	15.12
*α*‐helix / *β*‐sheet	1.06	2.15	2.15

To quantify the color, we measure L* values using a CIE spectrophotometer, where L* = 0 is absolute black, L* = 100 is absolute white, and L* < 5 corresponds to %R_avg_ < 0.5% for these particular wool fabrics [32]. AmW and PDAmW have similar initial L* of ∼13; however, after plasma etching, AmW and PDAmW achieve an L* ∼ 4.96 and L* ∼ 0.66, respectively, with PDAmW resulting in a more profound ultrablack effect (Table 1). Figure 1a‐fii provides a visual representation of the color of mW, PDAmW, AmW, and their plasma‐treated counterparts. Furthermore, PDAmW exhibits monotonic, featureless absorption in the visible region (400–800 nm) extending to the short‐wavelength NIR (800–1800 nm) region (Figure S3) [45, 51]. In contrast, AmW displays a reddish hue as a result of a steep reduction in absorption at ∼600 nm (Figure S3). Therefore, PDAmW enables the fabrication of ultrablack material due to both its enhanced and uniform etchability and wide range of optical absorption. The monotonic absorption across a wide wavelength range of PDAmW also facilitates photothermal applications [22, 32].

We further hypothesize that the initial color produced with PDA dyeing influences the final ultrablack effect; as such, we produce a series of colors, from grey to deep black with L* = 40 to 11, by varying the dyeing time (5 to 40 min) (Figure 1h,i). As expected, plasma‐etched pristine mW does not yield any noticeable optical effect (Figure 1d ii). Upon plasma etching PDAmW, L* values decrease across all samples; however, the final color depends strongly on the initial color, confirming the interplay between PDA dyeing and plasma etching. We observe three distinct regimes (Figure 1i): in region A, the grey color samples (L* > 40) produce no measurable improvement in L*; in region B, L* progressively decreases resulting in an increasing ΔL*; and in region C, the initial color is sufficiently dark (L* < 15), leading to saturation of the final L*∼ 0.55–0.65. The reduction in L* and the weight gain attributed to PDA uptake are found to be complementary; increasing PDA uptake decreases L*. Therefore, plasma etching enables the ultrablack limit to be approached, while the final color depends on the initial color achieved by dyeing. In region C, however, weight gain continues without a corresponding decrease in L*, which suggests that additional dyeing no longer deepens the color, but rather begins to contribute to film thickness (Figure S4).

These findings underscore the importance of dyeing optimization to achieve the darkest initial color. For polymerizing DA, we utilize SP, a two‐electron oxidizing agent; an acidic oxidative route is particularly well‐suited to dyeing wool. The concentrations of DA and SP, and their molar ratio, are found to influence dye uptake. An equimolar ratio of DA and SP produces the darkest and most uniformly colored PDAmW (Figure 1j). Excess SP leads to rapid PDA aggregation and irregular dyeing, while insufficient SP results in slower polymerization and a lighter coloration. With respect to solvent, ethanol‐based systems confer exceptional dye bath stability, with the PDA solution remaining free of visible aggregation even after 2 h; nonetheless, no significant difference in dye uptake is observed relative to aqueous systems. Temperature, by contrast, has the greatest influence on dyeing kinetics. By increasing the temperature from 30°C to 70°C, PDA dyeing significantly accelerates, reducing the dye cycle from approximately 35 to 5 min (Figure 1k). Dyeing at 70°C, however, results in irregular and inconsistent coloration. Therefore, we identify the optimal dye temperature to be 50°C, which results in a uniform color, lower dye time (∼20 min), and L* ∼ 11, which is lower than those previously reported. For comparison, we provide a summary in Table S2 of the dyeing conditions and L* values reported in the literature [14, 52, 53, 54]. Unless otherwise specified, we perform all subsequent experiments at 50°C, with an equimolar DA: SP (1 g/L: 1 g/L) ratio in aqueous solvent.

### 2.2 Role of Wool Fiber Type on PDA Dyeing

**FIGURE 2:**
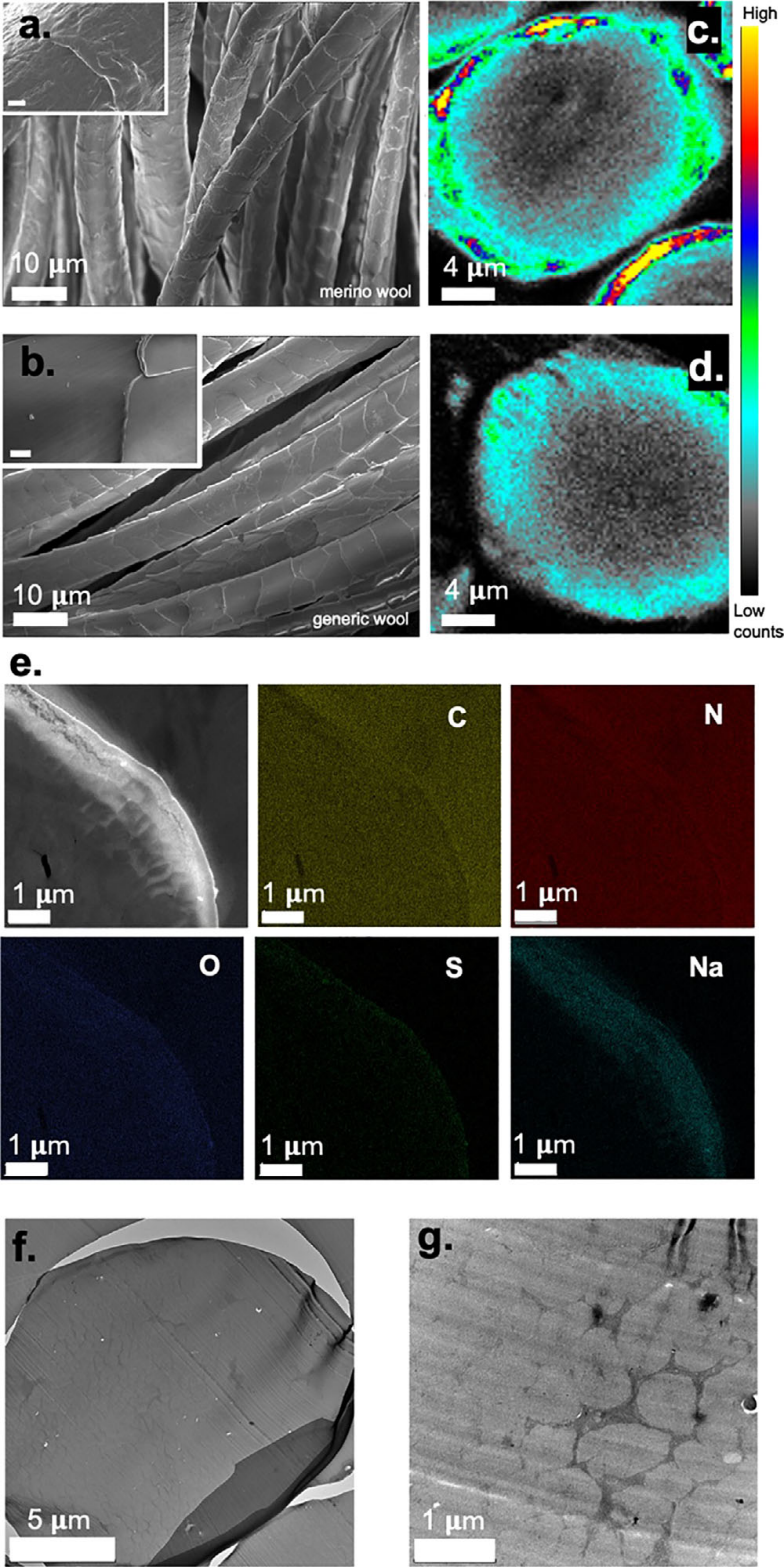
Effect of wool fibers on PDA dye uptake. SEM images of (a) merino wool fibers and (b) generic wool fibers; insets show higher magnification images of the cuticle scales (scale bars = 1 µm). 2D Raman maps of the graphitic G band of PDA (1580 cm^−^^1^) of cross sections of (c) merino wool and (d) generic wool fibers. (e) EDS spectra from HAADF‐STEM imaging showing PDA penetration and elemental mapping of a merino wool fiber cross section. Native ultrastructure observed by TEM of (f) PDA dyed and (g) undyed merino wool fibers.

Like many natural fibers, wool fibers exhibit hierarchical ordering, demonstrating a high degree of self‐organization across various length scales, from angstrom‐level protein sequence to microscale surface morphology. The structural and chemical features of the outermost cuticle layer as well as the internal cortex of the fibers determine the dye uptake. These variations are often greater among fibers of different diameters than between fibers of same diameter from distantly related species [39], therefore, for this study we compare two wool variants with distinct fiber sizes (Table 2; Figures 2a,b and S5); merino wool; a finer grade of wool harvested from the *Ovis aries* sheep (diameter, D ∼ 18.65 ± 3.32 µm) and a cheaper, and more commonly available generic wool with coarser fibers (D ∼ 24.40 ± 4.62 µm). The merino wool also corresponds to the mW fibers mentioned in the previous section. These finer merino wool is observed to have better dye uptake than generic wool (L* ∼ 11 and L* ∼ 13, respectively) (Table 2).

**TABLE 2:** Summary of chemical and structural data of the generic and merino wool.

	Generic wool	Merino wool
L*	12.47 ± 1.28	10.41 ± 1.19
Diameter, µm	24.40 ± 4.62	18.65 ± 3.32
Scales, 0.1 mm^−1^	∼8	∼6
*α*‐helix / *β*‐sheet	1.05	1.05
Contact angle, degrees	0	0

We first investigate the effect of surface chemistry and structure of the cuticle layer on the dye uptake. The cuticle, which comprises ∼10% of the fiber mass, is the protective outermost layer with an overlapping scale pattern. Wool is widely regarded as *α*‐keratinous; however, the cuticle is reported to contain a higher proportion of *β*‐sheets regardless of wool type [40]. The amide I bands in the FTIR spectra of the generic and merino wool, however, show strong *α*‐helix signatures and relatively low *β*‐sheet content, suggesting that the fiber diameter has minimal effect on protein conformation within this size range (Figure S6). Consequently, the effect of protein conformations on dye uptake cannot be assessed here. The SEM images (Figure 2a,b) of the fibers also show that both chosen wool types have regular coronal shaped scales. However, the generic wool has a higher scale density and sharper scale edges, while merino wool fibers are much finer with less frequent and smoother scales (Table 2). This indicates that the finer fibers with lower scale density yield better dye uptake, due to an increased surface area to volume ratio.

A recent work by Paul et al., however, contradicts this finding. They record the dye diffusion coefficient of Acid Violet 90 dyes by wool fibers; the finer fibers with lower scale densities resulted in reduced dye diffusion compared to coarser fibers [55]. Intercellular gaps between the scales are mentioned as the main diffusion pathway through which dye molecules penetrate the fiber structure. While the outer cuticle is relatively non‐reactive and hydrophobic due to its highly crosslinked structure and the outermost lipid monolayer, which is predominantly made up of 18‐methyleicosanoic acid (18‐MEA) [38]. Our findings suggest otherwise, with the diffusion pathway of PDA not being limited by the scales but occurring through intercellular gaps on the surface (high‐magnification SEM image provided in Figure 1a,b). High‐resolution XPS analysis (Table S3) also shows that the 18‐MEA lipid monolayer is altered, evidenced by the predominance of cysteic acid residues relative to thiol and thioester functionalities. As the latter groups are known to form covalent linkages with the lipid layer within the native keratin matrix, their reduction is consistent with the hydrophilicity of generic and merino wool we use for this study (Table 2). This is likely due to fibers being swollen in 50°C dye bath and damaged by a pretreatment process (e.g., scouring). Therefore, based on our findings, we conclude that the finer merino wool fibers have higher PDA dye uptake due to the higher effective surface area as well as the chemical and structural variations within the cuticle.

Dye penetration is also pivotal for creating an ultrablack surface, as the black colorant needs to be homogeneously distributed within the photonic architecture. This replicates the eumelanin nanogranules found within the keratin matrix of ultrablack feathers of Birds of Paradise (*Lawes’ Parotia*) [35]. Figure 2c,d illustrate the fiber cross sections of PDA dyed merino and generic wool captured by Raman spectroscopy. We report the 2D maps of the graphitic G band (1580 cm^−1^) intensity [56], which is attributed to PDA (Figure S7). The mapping portrays the penetration of PDA into the fiber structure, with a distinguishable peripheral bright band (width of ∼1 µm) and reducing in concentration toward the fiber cortex. High‐angle annular dark‐field scanning transmission electron microscopy (HAADF‐STEM) imaging and the corresponding energy‐dispersive X‐ray spectroscopy (EDS) also confirm the peripheral bright band surrounding the wool cortex, together with a pronounced contrast in the interior cortical region (Figure 2e). The prominent sodium (Na) in the PDA‐dominated band points toward association of Na‐containing species with PDA, while the presence of a bright sulfur band, which is attributed to the cystine‐rich keratin matrix of wool, further suggests strong interactions between PDA and sulfur‐rich species such as thiol and cysteic acid.

The annular intensity distribution observed in both 2D Raman mapping and STEM imaging is attributed to penetration barriers associated with the heterogenous wool ultrastructure. To further analyze this effect of wool ultrastructure on dye diffusion path, wool fibers are stained using uranyl acetate and imaged at higher spatial resolution using TEM. The cortex of native merino ultrastructure has a uniform matrix and clearly resolved polygonal microfibrillar network, where darker boundaries separate lighter domains (Figure 2f,g), consistent with literature on wool cortex hierarchy [57, 58]. For the PDA‐dyed merino wool (Figure S8), the overall cortical framework is retained, but two differences become evident: (i) a continuous darker peripheral band appears along the outer cortex area, and (ii) broader and more continuous dark inter‐domain pathways are seen, leaving the underlying keratin architecture largely intact. These observations corroborate the PDA peripheral‐augmented zone observed by 2D Raman mapping and STEM images. Furthermore, we identify the cell membrane complex as an additional diffusion pathway for PDA into the fiber interior, as validated by the lighter and darker intercellular interfacial regions observed in the STEM and TEM images, respectively. In HAADF‐STEM images (Figures 2e and S8), the cross‐section of dyed merino wool has a more continuous and thicker bright peripheral band together with a comparatively uniform outer cortical zone, while generic wool exhibits a less continuous and thinner rim, with greater local variability in cortical contrast. These differences are understood in terms of wool ultrastructure; fine merino fibers are known to possess a more clearly developed bilateral ortho‐ and para‐cortical organization, whereas coarser wools generally portray greater structural heterogeneity [59]. These findings underscore the intricate relationship between fiber diameter, cuticle and cortical structures, emphasizing the complex interplay that defines the hierarchical organization of keratinous fibers and the resulting heterogeneous dye diffusion pathways for PDA dyeing.

### 2.3 Role of Fabric Construction on PDA Dyeing and Optical Phenomena

**FIGURE 3:**
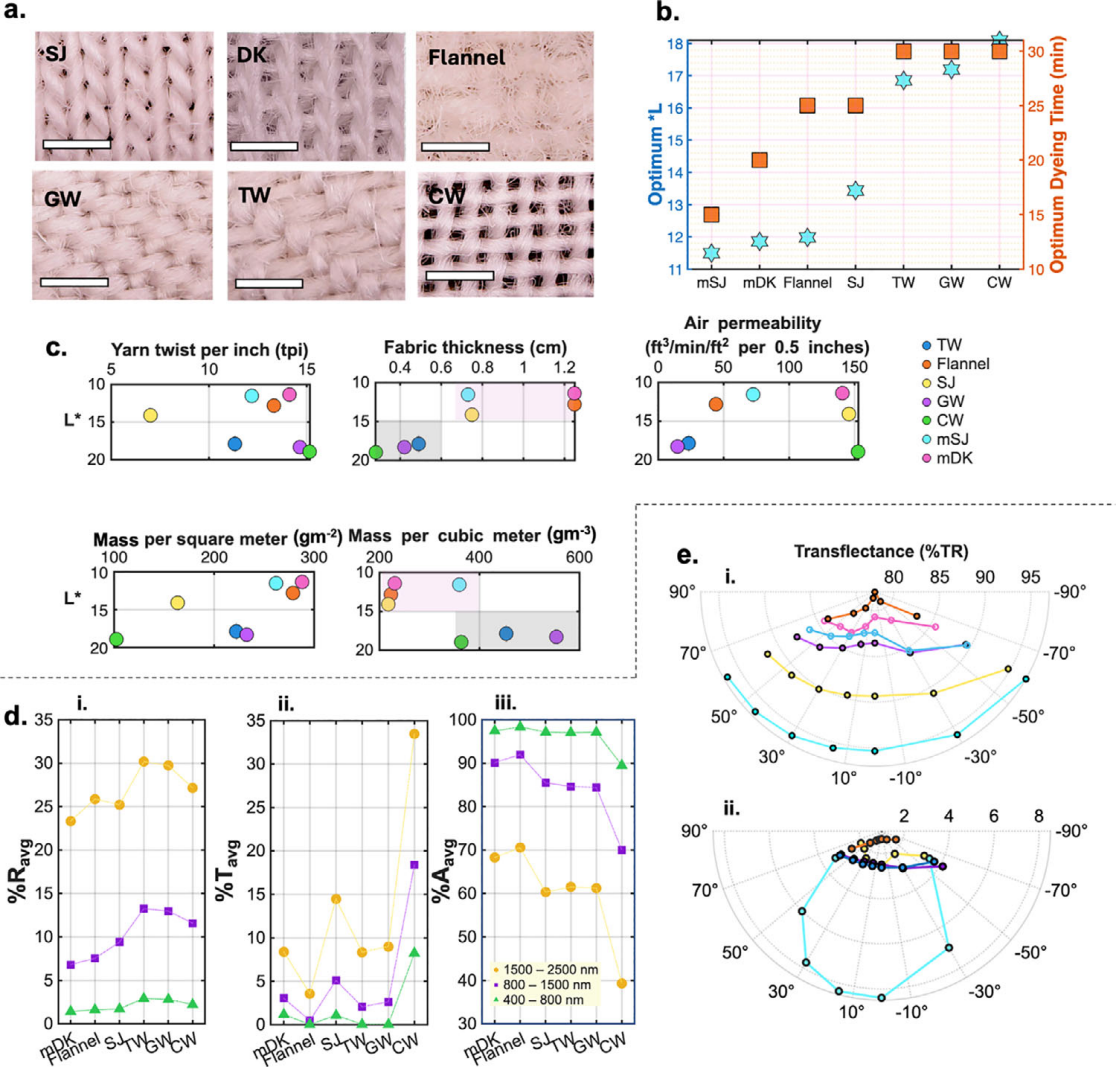
Effect of wool fabric architecture on PDA dyeing. (a) Close‐up photographs of different fabric constructions. The scale bar is 1 mm. (b) optimum L* and dyeing time for the different fabric constructions. (c) Effect of fabric properties on L* values. (d) effect of fabric construction on i. average total reflectance (%R_avg_), ii. average transmittance (%T_avg_), and iii. average absorbance (%A_avg_). (e) Hemispherical transflectance (TR%) of **i**. undyed wool fabrics and ii. PDA dyed wool fabrics.

The structural geometries of fibers and fabrics significantly affect the hydrodynamic boundary layer, which is the boundary surrounding the structure where the diffusion of dye molecules shows rapid deceleration and ultimately influences the overall dye uptake [60]. We compare fabrics: merino single jersey (mSJ), single jersey (SJ), merino double knit (mDK), flannel basket weave (Flannel), gaberdine weave (GW), twill weave (TW), and challis weave (CW) (Figure 3a), which have distinct fabric constructions and packing densities of yarns and fibers (Table S4). We summarize the optimum L* values and dye time for each of the fabrics in Figure 3b. Regardless of the construction, merino wool fabrics (mSJ and mDK, fiber diameter ∼ 18 µm) perform the best, achieving L* ∼ 11 in 10–15 min, whereas the tighter weave structures composed of generic wool, TW and GW achieve L* ∼ 17 at 30 min. CW, which has the highest porosity of generic wool fabrics, has the least barriers for dye uptake; however, it shows the highest L* ∼ 18 at 30 min (Figure 3b). To better understand this conflicting finding, we report the average total reflectance (%R_avg_) in the visible region (400–800 nm) (Figure S9); CW shows lower %R_avg_ than GW and TW, suggesting greater PDA uptake. The conflicting L* can be ascribed to residual transmittance (%T) from the open fabric structures. The progression of color with dyeing time (Figure S10), therefore, depicts the effect of structure on dye uptake and dyeing time, where all fabrics show no improvement beyond the optimum dyeing times.

To further identify parameters that drive PDA dye uptake, we plot fabric properties against L* (Figure 3c). Twist per inch, grams per meter squared (gsm), yarn count, and air permeability show no significant correlation with dye uptake, though greater fabric thickness correlates with lower L*. To account for the combined effect of material quantity and spatial distribution, grams per cubic centimeter (gcm) is also plotted against L*, assuming the fabric occupies a perfect cubic volume. While gsm alone shows no trend, gcm does portray one. When a unit volume contains less fiber mass, dye uptake is higher, consistent with a reduced diffusion barrier. We provide Pearson correlation matrix analysis in Table S5; results confirm the strong positive correlation between gcm and L* and negative correlation between thickness and L*. Given the limited number of data points, the matrix analysis is used as a validation tool rather than for formal statistical inference.

In addition to dye uptake, the fabric architecture, including fiber size and fiber‐yarn spatial arrangement, also affects the material‐light interactions [61, 62]. %R, %T, and absorbance (%A) measurements are used to monitor these optical effects. Unlike most commercial black dyes, PDA absorbs into the NIR region, which is relevant for photoconversion applications. We therefore analyze measurements across 400–2500 nm range in three spectral windows: (i) visible (400–800 nm), (ii) short‐wavelength NIR (SWNIR, 800–1500 nm), and (iii) long‐wavelength NIR (LWNIR, 1500–2500 nm) (Figures 3d and S9). The visible region captures absorption via chromophoric species, PDA in this case. The SWNIR domain contains higher‐order overtones of C─H stretching and the first overtones of O─H and N─H bonds, resulting in relatively weaker absorption, while the LWNIR captures the first overtones of C─H stretching and the stronger combination bands, which arise predominantly from ─CH, ─NH, and ─OH groups in wool chemistry [63]. We summarize %R_avg_ across these three regions for PDA‐dyed versus pristine wool in Table S6. PDA dyeing produces greater than 90% reduction in %R_avg_ in the visible region, compared to 75% reduction in the SWNIR, and a marginal reduction of less than 25% in the LWNIR (Figure 3d and Table S6). Structural deformations and increased porosity introduced during dyeing do not confound these measurements, as %T is also reduced across the full spectral range at a comparable rate (Figure S11 and Table S6).

In the visible region, %R_avg_ is predominantly dictated by chemical absorption by PDA, whereas the fabric structural influence becomes minimal (Figure 3d). In contrast, in the SWNIR and LWNIR regions, the effect of fabric structure on %R_avg_ becomes prominent, where the influence of dye absorption diminishes, and the measured reflectance increasingly shows overtones and molecular vibration bands characteristic of keratin. For %A_avg_, however, fabric structure is important across the entire Vis‐NIR range as %T_avg_ is significantly affected by structural parameters. For photoconversion device applications using PDA‐wool textiles, fabric structure therefore plays a critical role and can be optimized depending on whether %R or %A is the more performance‐critical parameter.

We also report optical behavior by measuring the transflectance (%TR) at varying angles of incidence (i.e., 60° to +60°) for both pristine and PDA‐dyed fabrics (Figure 3e). %TR measures the combined optical response of both reflectance and transmittance by positioning the sample at the center of an integrating sphere and varying its angle of incidence. For pristine fabrics, irrespective of the fabric structure, the %TR response change is symmetrical and minimal, with an increase in %TR of 1%–7% across the angular range (Figure S12). After PDA dyeing, the symmetry is retained; however, for higher porous structures (those with greater air permeability and %T), %TR decreases at higher angles of incidence as the light grazes more material at oblique angles, reducing the transmittance significantly compared to 0°. For tightly woven structures like GW and TW that inherently possess low transmittance, an increase in %TR at higher angles of incidence (relative to 0°) is driven by an extended optical path length, which proportionally enhances reflectance. These findings confirm that fabric structure has direct implications for wide‐angle optical performance. For the successful creation of optimal fabric‐based optical devices, it is therefore necessary to engineer fabric structures with attention to both its effect on dye uptake and light‐material interactions.

### 2.4 Mechanistic Insights Into DA Polymerization and Wool‐PDA Interactions

While multi‐level hierarchical structure influences the wool dyeing process, understanding the fundamental mechanistic pathways is required to elucidate the distinct driving forces of PDA dyeing as opposed to surface coating, a distinction that is critical for achieving ultrablack textiles. Contrary to dyed PDA fabrics, plasma etching of a PDA‐coated fabric yields a progressively brighter surface rather than a darker one [32]. Therefore, a mechanistic understanding of PDA polymerization and PDA–wool interactions may also guide the extension of ultrablack textile fabrication to other natural fibers such as cotton or silk.

**FIGURE 4:**
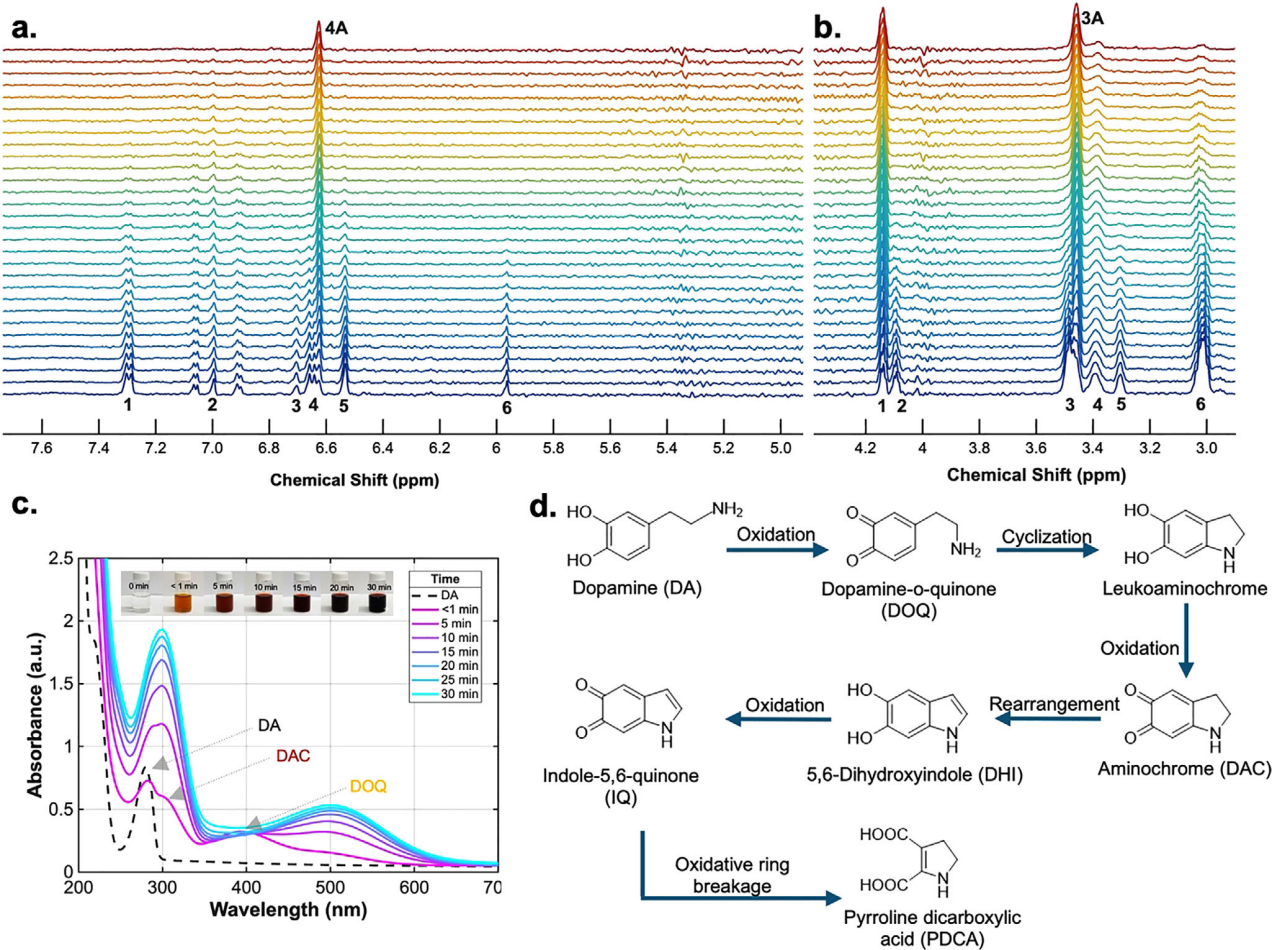
Temporal evolution of dopamine‐derived intermediates during PDA dyeing. ^1^H NMR spectra illustrating temporal evolution of species in PDA dye bath (a) aromatic region, (b) aliphatic region. (c) Temporal studies of the PDA dye bath using UV–vis spectroscopy. Inset shows color change of solutions observed visually with time. (d) Proposed early‐stage oxidative transformation pathway in the periodate‐mediated dye bath.

First, we monitor the evolution of dopamine‐derived intermediates in the dye bath by NMR and UV–vis spectroscopy over 0–30 min using mSJ fabric at 50°C with an equimolar DA: SP ratio (Figure 4a–c). Time‐resolved proton NMR spectra are acquired every 1 min after the addition of SP (Figure 4a represents the aromatic region of the spectra, while *b* represents the aliphatic region). We also record complementary UV–vis spectra in the region of 200 to 700 nm immediately after the addition of SP and at subsequent 5‐min intervals. The DA spectra obtained prior to SP addition show the characteristic catechol aromatic protons and aminoethyl side‐chain methylenes (Figures 4c and S13), confirming DA as the only NMR‐visible species present at the start of the reaction. The characteristic DA catecholamine peak at ∼280 nm confirms the same from the UV–vis spectrum (Figure 4c).

We identify the dominant absorbing species in our system using a multiple species matrix analysis of UV–vis data, which is explained by Graham et al. [8]. This confirms the presence of at least three distinct species (Figure S14): (i) DA (280 nm), (ii) dopamine‐o‐quinone (DOQ; 395 nm) and (iii) dopamine aminochrome (DAC; 302, 480 nm). These species are also visually identified by a color change in solution; DOQ is yellow, DAC is red, and the rapid conversion to orange at the onset of the reaction results from the co‐existence of DOQ and DAC (Figure 4c *inset*). An isosbestic point near ∼410 nm is consistent with the formation and conversion of DOQ to DAC; however, it is not strictly maintained. The duration over which the isosbestic point is observed decreases with increasing temperature, emphasizing that these transitions are temperature and concentration sensitive. The faster generation of DOQ at 50°C allows for efficient unimolecular cyclization to DAC, and potentially subsequent conversion to indole‐type units which cannot be traced using UV–vis due to overlapping signatures (5,6‐dihydroxyindole (DHI) peak appears at 320 nm [9, 64]).

The in situ NMR spectra reveal that DA polymerization proceeds through multiple spectrally and kinetically distinguishable, overlapping intermediates rather than a one‐step transformation (Figure 4a,b). The aromatic and aliphatic sections in the NMR spectra are both divided into 6 regions, and their temporal evolutions are presented in Figure S13. In the aromatic section (Figure 4a), region 1 is downfield (∼7.3 ppm) and exhibits rapid and monotonic decay (Figure S13); thus it is assigned to a short‐lived quinonoid aromatic environment, most plausibly DOQ. We also observe this briefly in the UV–vis spectrum at ∼395 nm. Interestingly, region 2, an overlap region, consists of residual DA together with emerging oxidized/cyclized products, as the corresponding temporal change does not display a simple one‐component decay. This indicates transient accumulation of an intermediate in a consecutive reaction sequence, but the clearest signature of transient cyclized intermediate populations is in region 3–5 (∼6.7–6.5 ppm). Within this, region 4 (specifically 4A) delineates the obvious rise and fall behavior of an intermediate species, and is ascribed to aminochrome‐rich species, consistent with literature [65, 66, 67]. This matches UV–vis data, where there is a rapid fall and rise of peaks in the 280–305 nm region. Region 3 and 5 carry some early DA overlap, while region 5 also represents the lower‐ppm edge of the same cyclized manifold. At the same time, minor late‐stage overlap from downstream indolic/oligomeric species cannot be excluded, particularly in region 5. Since DHI includes an indolic proton in the ∼6.1–6.0 ppm range [68], region 6 is best assigned to DHI‐like indolic species, with possible later overlap from downstream products like indole‐5,6‐quinone (IQ).

The aliphatic section of the spectra (Figure 4b) independently echoes similar intermediates and sequence of oxidative polymerization. The most downfield aliphatic signals (region 1 and 2) are deshielded, and therefore, we assign them to an early oxidized/cyclized side‐chain environment. Importantly, region 2 itself decreases with time, whereas the combined region 1 + 2 envelope displays rise‐and‐fall behavior (Figure S13). This indicates that the two integration windows do not represent distinctly resolved individual species, but rather capture different parts of the same evolving downfield aliphatic signature. We interpret these two regions as encompassing DOQ‐derived and/or leukoaminochrome‐like aliphatic environments that redistribute rapidly as cyclization proceeds. This agrees with the aromatic data, where the quinonoid behavior is likewise restricted to a short‐lived downfield region before the cyclized species dominate. Regions 3–5 are attributed to the cyclized intermediate population, resonating with the aromatic assignment of regions 3–5 as aminochrome‐rich. Specifically, the transient species in 3A is intriguingly similar to the intermediate population in aromatic region 4A, highlighting complementary aliphatic and aromatic signatures. Within these regions, a minor overlap from residual DA is possible at early times and from low molecular‐weight DA‐derived dimers/oligomers at later stages. Region 6 is the most DA‐like aliphatic signal and primarily demonstrates the monotonic decay signature as expected.

Using the in situ NMR and UV–vis analysis, we propose a mechanistic pathway for the oxidative polymerization in our system (illustrated in Figure 4d), where DA first undergoes rapid oxidation to quinonoid species (DOQ), followed by intramolecular cyclization and oxidation to leukoaminochrome and aminochrome‐rich intermediates. There are subsequent rearrangements and conversion to downstream indolic products such as DHI and IQ, which is then succeeded by low‐molecular‐weight oligomers and incipient PDA aggregates. Consistent with past studies, our spectra point toward overlaps from oligomer resonances in both aromatic and aliphatic areas, but distinct oligomer signatures cannot be deciphered because of strong congruence with other signals, non‐accumulation to NMR‐detectable concentrations and/or rapid broadening/precipitating as the system progresses toward PDA through oligomer growth, aggregation, and supramolecular assembly [45, 65].

To better understand the bath‐to‐fiber transfer, reaction baths in the presence and absence of wool are compared by aliquot‐quenching experiments using sodium thiosulfate as the quenching agent. Sodium thiosulfate rapidly consumes residual periodate/iodate oxidant [69], thereby inhibiting further oxidation after sampling. Samples are taken out every 10 min, and the corresponding proton NMR spectra are shown in Figure S15. Even though DA signatures are consumed in both cases, they disappear far more rapidly in the presence of wool. This conveys a dynamic bath‐to‐fiber transfer of reactive intermediates through coupled oxidation and dyeing, complementary to a recent study that analyses PDA adsorption on halloyside nanotubes [47]. Looking closely at the temporal evolution of species, the faster generation of DOQ allows for efficient cyclization to DAC; at the initial stage of up to 20 min, which neatly overlaps with the optimum dyeing time of the majority of wool textiles used for this study. The DOQ is also chemically reactive and can attack nucleophiles such as sulfur‐rich species before it cyclizes to DAC [70]. We note, however, that an effective dyeing cycle can occur at any 15–20 min interval, within 60 min of initiating the polymerization (Figure S16), suggesting that the PDA polymeric forms within the first 20 min are not the only species interacting with wool. These findings provide mechanistic insights into PDA dyeing; under the conditions examined, dyeing appears to be predominantly governed by the absorption and in situ fixation of intermediate species within the keratin matrix. This contrasts with the solubility‐controlled aggregation, deposition, and surface adhesion model commonly invoked in the literature to describe PDA coating formation [71].

**FIGURE 5:**
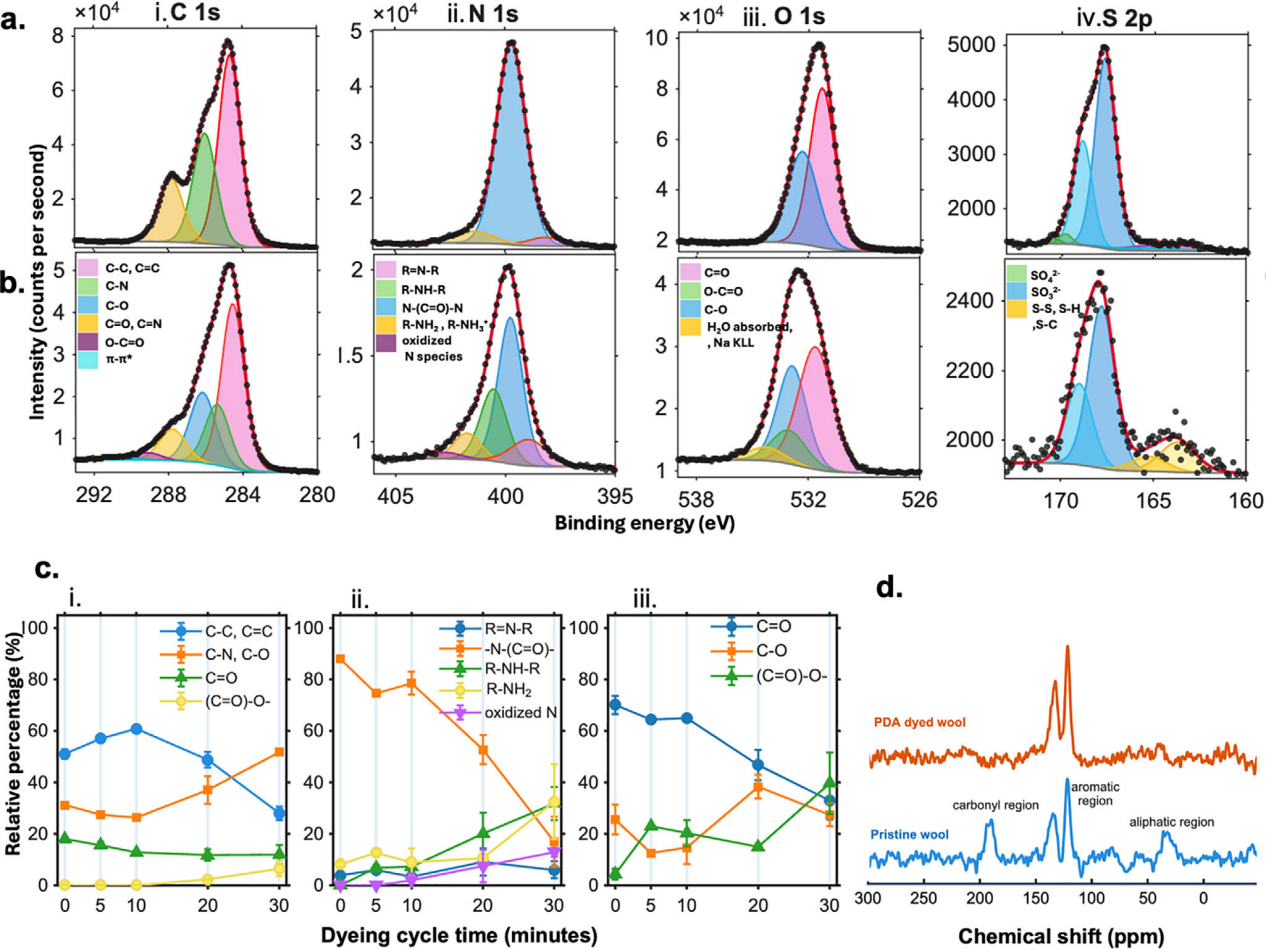
Wool—polydopamine interactions. High‐resolution XPS spectra of C 1s, O 1s, N 1s, and S 2p of (a) pristine wool and (b) PDA dyed wool (dyeing cycle time of 20 min). (c) Temporal evolution of PDA‐wool species identified from deconvoluted high‐resolution XPS spectra and (d) ss carbon NMR spectra of pristine wool and PDA dyed wool (dyeing cycle time of 20 min).

To understand the evolving wool surface chemistry during PDA dyeing, we probe the outermost 5–10 nm of the fiber using XPS. A SP‐treated wool control confirms that the oxidant alone produces no measurable changes in the C 1s, N 1s, O 1s, or S 2s spectra relative to untreated wool (Table S7), and all spectral changes during dyeing are therefore attributed to PDA‐wool chemistry (Figure 5a,b). In pristine wool, the spectra reflect the keratin polypeptide: predominantly aliphatic C─C, C─H, and C═C environments in the C 1s, dominant keratin amide nitrogen in the N 1s, and backbone carbonyl with aliphatic C─OH from side chains (serine, threonine, and tyrosine) in the O 1s (Figure 5c). The most consistent observation across the temporally evolving spectra is the progressive displacement of these keratin signals with PDA relevant species. The survey‐level atomic composition verifies this macroscopic surface transformation (Table S7). Carbon increases monotonically with dyeing time while the N/C ratio converges toward approximately 0.11 after 30 min (Figure S17). This value is below the idealized theoretical value of 0.125 for the C_8_H_7_NO_2_ indole‐type moiety, which is an expected consequence of oxidative nitrogen loss under excess periodate as reported by Ponzio et al. [64]. The sulfur signal, from cystine and cysteine residues of the keratin matrix, attenuates progressively and becomes unquantifiable by 30 min as the PDA film coats the underlying keratin matrix. This is consistent with the weight gain data (Figure S17), where the majority of mass uptake occurs within the first five minutes. Additional surface deposition beyond 20 min, once accessible reactive sites are saturated, reflects a transition from preferential molecular penetration to surface‐localized PDA coating, beyond which the color does not evolve further (L* ∼ 11).

Alongside the attenuation of wool signatures, several spectral features unique to PDA chemistry emerge progressively (Figure 5b,c). At the earliest dyeing timepoints, the C─C, C─H component in the C 1s increases rapidly then declines, tracing a parabolic trend as DA monomers and oligomers enrich the keratin matrix, followed by attenuation of the peptide C environment. Concurrently, a nitrogen component at approximately 400.1 eV grows progressively in the N 1s. While this binding energy is consistent with primary amine nitrogen (─NH_2_), it may also encompass keratin amide (N─(C═O)─) as these two contributions are not fully resolved at this binding energy. Based on the resolved spectra, the primary and secondary amine fractions grow substantially as the peptide amide signal attenuates, suggesting the incorporation of N environments from uncyclized DA as well as aminoethyl‐bearing PDA oligomers such as DOQ and DAC‐type cyclized products. The preservation of free amine groups at this stage is consistent with DA moieties observed via solution‐state NMR and UV–vis spectroscopy. In the O 1s, aliphatic C═O decreases sharply as phenolic catechol C─OH becomes dominant, albeit showing a sigmoidal trend, as the PDA progressively undergoes structural changes. As dyeing progresses, a carboxylate contribution absent in keratin appears in the C 1s, indicating aromatic ring opening of catechol units through excess periodate to yield fragments such as pyrroline dicarboxylic acid (PDCA) (Figure 4e) [64]. Concurrently, *π–π* ^*^ shake‐up features of conjugated aromatic systems become resolvable, consistent with the presence of aromatic chromophore characteristic of PDA and compatible with the *π–π* stacking interactions widely reported for PDA assemblies [72]. This shake‐up satellite, nevertheless, does not constitute direct structural proof of intermolecular stacking by itself. A feature coinciding with the Na KLL Auger emission appears in the O 1s, which is consistent with Na^+^ co‐diffusion alongside penetrating PDA species, as corroborated by HAADF‐STEM‐EDS mapping (Figure 2e). The imine and quinone‐imine components in the N 1s fluctuate non‐monotonically across the time series, in agreement with the slight accumulation of tautomeric DAC and indolequinone intermediates, though its low absolute contribution requires caution and may account for spectral noise. Collectively, the spectral evidence points to a chemically heterogeneous population of species within the wool matrix, ranging from uncyclized DA species and early aminoethyl‐bearing oligomers in the initial stages, through tautomeric DAC and indolequinone intermediates, to π‐stacked PDA assemblies and oxidatively ring‐opened carboxylate fragments at later timepoints.

The persistence of sulfur environments throughout the active penetration window implies that cysteine and cysteic acid remain accessible as potential covalent anchoring sites for PDA. XPS data alone, however, does not provide concrete evidence of the exact covalent and/or noncovalent interactions that drive wool‐PDA association. Solid‐state NMR spectroscopy of PDA‐dyed wool provides some insights into these potential interactions. The carbon environments of both pristine and PDA‐dyed wool are compared in Figure 5d, where characteristic keratin signals are present in the pristine sample with well‐defined aromatic, carbonyl, and aliphatic regions [73]. The attenuation of the keratin carbonyl and aliphatic signals in PDA‐dyed wool implies that a fraction of those sites now resides in a PDA‐rich interphase, where NMR observability is reduced through changes in local dynamics and relaxation. Since PDA contains persistent semiquinone‐type radicals, and paramagnetic species are well known to shorten relaxation times and attenuate nearby solid‐state NMR signals [74, 75], the reduced carbonyl and aliphatic intensities are consistent with close spatial proximity of these keratin segments to PDA domains, producing restricted mobility and faster relaxation of the associated keratin nuclei. These perturbations are attributable to hydrogen bonding between catechol and indole‐derived OH and NH groups of PDA and the amide carbonyl and side‐chain hydroxyl groups of keratin. The enhanced aromatic signal in PDA‐dyed wool is consistent with the incorporation of a PDA‐rich phase and is compatible with *π–π* stacking at the wool‐PDA interface. The proton environment results are presented in Figure S18, where PDA‐dyed wool exhibits a more localized solid‐state NMR signal, indicating that dyeing generates a distinct population of strongly deshielded protons relative to the broader distribution observed for pristine wool.

These mechanistic studies imply that the periodate‐mediated PDA polymerization under the explored conditions promotes multiple reactive species to penetrate and engage covalently and noncovalently with the keratin matrix within ∼1 h of initiation. Thereafter, PDA aggregate formation is favored, and surface coating predominates over dyeing.

## 3 Conclusion

We leverage the eumelanin‐like photon absorption and adhesion properties of PDA to provide an effective route to optically functional textiles, including ultrablack wool. Using excess SP at 50°C, we substantially reduce the dyeing time of wool fabrics from those reported in the literature to within 30 min, with some merino variants reaching optimal color in as little as 15 min. Beyond accelerated dyeing, we delineate the structural and mechanistic pathway of PDA formation during textile dyeing.

At the multiscale structural level, PDA incorporation in wool is governed by keratin organization, fiber ultrastructure, surface chemistry, and the fiber‐yarn‐fabric hierarchy. Fiber diameter and structural parameters are closely interrelated, whereby finer fibers, with correspondingly higher surface areas, show higher dye uptake than their coarser counterparts. Cuticular chemistry and structural order also exert measurable influences. Fabric architecture strongly influences dye transport, with fabrics of lower volumetric density showing higher uptake, while also governing the broadband wavelength optical response across a wide angular range. Additionally, we found that fabric structures with fibrous napped surfaces may also improve dye uptake and affect light‐surface interactions. Therefore, understanding the precise role of surface roughness and fabric spatial arrangement on light interaction could be an interesting future study.

Lastly, in this work, we propose a pathway for PDA formation in our system, where the dyeing initiates with rapid absorption of DA and DAQ‐like intermediates, with fabrics gaining approximately 8 w/w% within the first five minutes. A uniform ∼1 µm radial penetration band spanning the cuticle‐cortex interface transitions to an annular gradient in the fiber interior, consistent with preferential dye penetration through the intercellular matrix, distinguishing this absorption‐driven mechanism from aggregation and adsorption/deposition driven pathways reported in the literature. These temporal observations suggest molecular absorption dominates at early stages, transitioning to surface film formation at later stages. The precise covalent coupling between DA monomeric/oligomeric species and keratin reactive sites such as thiol groups warrants further investigation. Furthermore, quantitative studies such as kinetics of diffusion, polymerization kinetics, and temporal thickness profiles across the fiber cross‐section would be interesting directions for future work.

Collectively, these findings provide a mechanistic design principle for PDA‐functionalized textiles in photonic applications, with dopamine chemistry, fiber structure, and fabric architecture emerging as vital parameters in controlling the dye uptake for wide‐angle optical performance. More broadly, the insights provided in this study have implications across multiple fields including engineering biomaterials, surface science, nanotechnology, and energy materials.

## 4 Experimental Section

### 4.1 Materials

Single jersey merino wool (260 g/m^2^), acid black dyed single jersey merino wool (260 g/m^2^, exact dye identity is unknown), and double knit merino wool (285 g/m^2^) fabrics were purchased from Nature's Fabric, USA. Twill woven (220 g/m^2^), flannel basket woven (280 g/m^2^), single jersey (165 g/m^2^), gaberdine woven (230 g/m^2^) and challis woven (100 g/m^2^) were purchased from Testfabrics.Inc, USA. Dopamine hydrochloride (≥98%) and sodium periodate (≥99.8%) were purchased from Sigma–Aldrich. All materials and chemicals were used as received.

### 4.2 PDA Wool Dyeing

To determine optimal dyeing conditions, the effects of concentration, molar ratio, temperature, dyeing time, and solvent system were systematically investigated. Solutions of sodium periodate (NaIO_4_;SP) and dopamine hydrochloride (DA) were prepared at DA concentrations of 1 and 2 g/L, with DA:SP molar ratios of 1:1, 2:1, and 4:1. Pristine merino wool fabrics were immersed in the dye solutions at a material‐to‐liquor ratio of 1:100 and continuously agitated. Dyeing was carried out at temperatures of 30°C, 50°C, and 70°C for durations ranging from 5 to 60 min. The solvent system was also varied; in addition to 100% aqueous solution, ethanol–water and methanol–water mixtures at 25% and 50% co‐solvent concentrations were evaluated. Following dyeing, all fabric samples were rinsed in water and air dried. Circular fabric specimens of 38 mm diameter were prepared using a fabric sample cutter (SDL Atlas) for subsequent optical measurements.

### 4.3 CIELAB Spectrophotometer

Colorimetric data were measured using a Ci7800 Sphere Benchtop Spectrophotometer. The instrument simultaneously measures spectral reflectance from 360 to 750 nm at 10 nm intervals. The aperture diameter of the measuring port was 17 mm. Color coordinates were measured according to the CIE L*a*b*. Illuminating and viewing conditions were chosen to be CIE diffuse/8° geometry.

### 4.4 Scanning Electron Microscopy (SEM)

The field emission scanning electron microscopy (FE‐SEM) (Zeiss Gemini500 FE‐SEM) was used to image wool samples after sputter coating with gold‐palladium for 40 s. For optimum imaging, an acceleration voltage of 1.5 kV was used. Fiber diameter and scale density were measured using SEM images by using ImageJ.JS V0.6.0 software.

### 4.5 Raman Spectroscopy

Several fibers were bundled tightly in Whatman #1filter paper, making a sheath to hold the fibers together. An adhesive, LockTite 380, is then placed on top of the bundle so that it is absorbed into the filter paper and the fibers. The sample is then left to harden, 1 h minimum at room temperature. The polymerized bundle is then scraped off the microscope slide and placed into a suitable sample holder for the Leica Ultracut 7n Ultramicrotome. The sample is sectioned dry on a Diatome 35‐degree diamond knife with several passes in 500 nm increments until a smooth, uniform area is achieved. A confocal Raman microscope (WiTec Alpha300R) with 532 nm laser was used to analyze the fiber cross‐sections. 2D maps and line scans were collected with an integration time of 1s and accumulations of 50 and 3 for line scans and 2D maps, respectively. Line plots and 2D profiles were collected for 1350 and 1580 cm^−1^ bands corresponding to disordered D and graphitic G bands of PDA. In order to facilitate comparison between merino wool and generic wool, instrument parameters such as laser power, integration time, and the number of scans were kept consistent across samples.

### 4.6 High‐Angle Annular Dark‐Field Scanning Transmission Electron Microscopy (HAADF‐STEM) and TEM

The fibers were soaked in 100% EMBED812 epoxy resin for 48 h on a rotor so that the fibers were better infiltrated with the epoxy. After the infiltration, the fibers were moved to a silicone flat embed mold and placed carefully to facilitate easy cross‐sectioning. The samples were topped off with EMBED812 and moved to a 60‐degree Celsius curing oven for polymerization for 12–24 h. The polymerized samples were cross‐sectioned using a Leica UltraCut 7 ultramicrotome. A 35‐degree Diatome diamond knife was used to make the 70 nm‐thick cross‐sections in water. The cross‐sections were then retrieved from the water on 3 mm single‐hole slot grids coated with polyvinyl Butvar.

Images and analysis were done using a Thermo Fisher Scientific Spectra 300 in HAADF and TEM modes at 120 kV. The X‐ray data are acquired with Bruker DualX detectors. The staining for TEM was carried out using uranyl acetate.

### 4.7 Fourier‐Transform Infrared (FTIR) Spectroscopy

The functional group analysis was conducted using an attenuated total reflectance Fourier‐transform infrared (ATR FTIR) spectrometer (PerkinElmer). The spectra were recorded in the range of 4000–800 cm^−1^ at a resolution of 4 cm^−1^, and 32 scans were conducted for each spectrum. The amide I region (1700 − 1600 cm^−1^) was further analyzed using Origin Pro 2026 by deconvoluting the peak to capture the content of secondary protein conformations. To ensure accuracy of fitting, the second derivative function of the peak is plotted, and peaks are assigns accordingly. Peaks are then fitted by first performing baseline corrections, smoothing using Savitzky‐Golay (13 frames), and Gaussian functions.

### 4.8 X‐Ray Photoelectron Spectroscopy (XPS)

X‐ray photoelectron spectroscopy (XPS) spectra were collected using a Nexsa G2 Surface Analysis System (ThermoFisher Scientific) using a focused Al K monochromatic X‐ray source (1486.6 eV). All XPS spectra were analyzed using the CasaXPS software (version 2.3.26) with binding energies charge‐corrected to the C 1s peak set at 284.8 eV. Atomic concentration (%) was calculated using high‐resolution scans, and peak fitting was performed using GL(30) and FWHM ∼0.8–2.5 as appropriate for different species while maintaining RSF close to 1.00. XPS database provided by Thermo Fisher Scientific and other relevant literature were used to assign chemical species to resolved species.

### 4.9 UV‐Vis & UV–Vis–NIR Spectroscopy

#### 4.9.1 Diffuse Reflectance Accessory (DRA) Measurements

Total reflectance (%R) and transmittance (%T) were measured using an Agilent Cary 5000 UV–vis–NIR spectrophotometer with a 150 mm sphere integrating sphere (Cary External DRA) to capture total hemispherical radiation (8° tilt). Absorbance (%A) was calculated with a simple formula of %A = 100 − (%R + %T). The transflectance (%TR) was measured by mounting a clip‐style variable‐angle center‐mount accessory to the DRA. The sample was placed in the center of the integrating sphere facing the incident angle at the 0° position (i.e., normal incidence). As indicated on the top of the center mount module, turning counterclockwise represented a negative angle and turning clockwise represented a positive angle, resulting in the full range of −60° to 0° to 60°.

#### 4.9.2 Liquid State Measurements

To analyze polymerization kinetics of DA solution over time at different temperatures, the optical densities (absorbance) of the solution were measured using a UV–vis spectrophotometer (Shimadzu 2550). All solutions were diluted by a factor of 20 before taking measurements in the region 200–800 nm. As the instrument doesn't allow temperature control, solutions were quickly transferred from the dye bath, diluted, and measurements were taken at ambient conditions.

### 4.10 Nuclear Magnetic Resonance (NMR) Spectroscopy

All NMR data were collected at the Cornell University NMR Facility (RRID: SCR_028078). Specifically, 13C magic‐angle spinning direct‐polarization solid‐state NMR experiments were conducted using a Varian Unity‐Inova 500 spectrometer equipped with a 3.2 mm Phoenix MAS NMR probe and rotors, operating at 11.75 Tesla (500 MHz) to facilitate carbon quantification. Chemical shifts were measured by reference to tetramethylsilane (TMS, 0 ppm), and the magic angle setting to achieve high spectral resolution was performed using potassium bromide (KBr). The liquid NMR measurements, in situ and aliquot quenching ones, were conducted using 600 MHz Varian INOVA and 500 MHz Bruker AVIII HD spectrometers, respectively.

### 4.11 Fabric Property Analysis

Twist per inch of yarns was measured using a twist gauge (Emerson apparatus Co.). Air permeability was measured using a porometer, and the fabric thickness was measured using a mechanical fabric thickness gauge by setting the pressure footer to apply zero pressure. Hydrophilicity of fabrics was measured using a contact angle goniometer (Ram Hart 500).

## Author Contributions

Conceptualization: H.J., L.S. optimization studies: H.J., M.B. characterization of materials: H.J., K.S., K.P. visualization: H.J. investigation: H.J., K.S. supervision: L.S. Writing – original draft: H.J., K.S. Writing – review & editing: H.J., K.S., L.S.

## Conflicts of Interest

The method of plasma treating wool to create an ultrablack textile is covered in below provisional patent. The authors declare the following competing interests: Patent Applicant: Cornell University. Inventors: Larissa M. Shepherd, Kyuin Park, and Hansadi Jayamaha. 11396‐01‐US. Filed Provisional Patent.These authors declare no other competing interests. All other authors declare they have no competing interests.

## Supporting information




**Supporting File**: advs77312‐sup‐0001‐SuppMat.docx.

## Data Availability

Raw data generated from this study can be accessed at https://doi.org/10.7298/9ws7-v764. All other data needed to evaluate the conclusions in this paper are present in the main text and/or the Supplementary information.
