# Supplementary material for: A Multiscale Mechanistic Framework for Polydopamine Dyeing in Ultrablack Wool Textiles
Source: Adv Sci (Weinh). 2026 Aug 24;13(54):e77312. doi: 10.1002/advs.77312 (PMC13502351; doi:10.1002/advs.77312)
Supplement: Supplementary file 1 [file ADVS-13-e77312-s001.docx]

Supporting Information

A Multiscale Mechanistic Framework for Polydopamine Dyeing in Ultrablack Wool Textiles

Hansadi Jayamaha^1^, Kumar Siddharth^1^, Kyuin Park^1^, Mia Bressler^2^, Larissa M. Shepherd^1*^

# **Figure S1****.**

# SEM images of plasma etched a. polydopamine dyed merino wool (PDAmW) fibers and b. acid dyed merino wool (AmW) fibers. The images show the difference in etching uniformity. Two magnification levels are provided in i. and ii for above fibers.

# **Figure S2.**

# FTIR of pristine merino wool (mW), PDAmW and AmW. a. full spectrum, b. secondary protein conformation peak assignments by deconvoluting the amide II band (1700 – 1600 cm^-1^).

# **Figure S3.**

# UV-Vis-NIR spectra of mW, PDAmW and AmW a. total reflectance, b. transmittance and c. absorbance

# **Figure S4.**

# SEM images showing temporal evolution of PDA coating on wool fiber surface. For 20 minutes, which is the optimum time for most wool fabrics dyed in our study, both a higher magnification and lower magnification image is provided to show the uniformity of the coating.

# **Figure S5.**

# SEM images showing the distinct scale geometries and density of a. generic wool and b. merino wool.

# **Figure S6.**

# FTIR of merino wool and generic wool. a. full spectra, b. second derivative of the amide I region and peak assignments *(42, 49)*. The second derivative is provided here to confirm the peak assignments.

# **Figure S7.**

# Raman spectroscopy of a. mW, b. PDAmW and c. PDA dyed generic wool (PDAW). Measurements are taken at edge of fiber cross-section to capture the highly concentrated PDA band at the periphery of the fiber surface. The I_G_/I_D_ ratio is ~1.78 for both PDAmW and PDAW. The amide II spectral region (1510–1580 cm⁻¹) of pristine wool also affects the I_G_ component.

# **Figure S8.**

# a. HAADF-STEM of PDA dyed merino wool and b. PDA dyed generic wool

# **Figure S9.**

# UV-Vis-NIR spectra of different fabrics after PDA dyeing a. total reflectance, b. transmittance and c. absorbance

# **Figure S10.**

# Progression of L* (lightness) with time for different fabric architectures

# **Figure S11.**

# Photographs of fabrics after dyeing showing the more disordered fabric architecture after dyeing.

**Figure S12.**

Changes in average transflectance (%TR) is plotted against the variable angle of incidence for a. pristine fabrics and b. PDA dyed fabrics. Delta %TR = (%TR - %TR_0_)/ %TR_0_ where %TR is the transflectance at normal incidence. The irregular spectra of mDK is attributed to deformed fabric architecture of mDK after PDA dyeing (refer Figure S11)

a.

b.

c.

**Figure S13.**

^1^H NMR temporal evolution studies a. dopamine spectra before addition of sodium periodate (SP). Temporal evolution of species after addition of SP b. aromatic region, c. aliphatic region.

**Figure S14.**

3-Species Matrix Analysis *(8).* Enforced conditions as per literature are;

1. i ≠ m : wavelength index i must not equal m,

2. j ≠ x, y, z : plotted tracings must differ from fixed reference tracings,

3. x, y, z are arbitrary but fixed real measured tracings (1, 10 and 30 min) and

4. j ≠ x, y, z leaves j=2 (5 min) and j=4 (20 min) as the plotted tracings

The fitted lines do not pass through the origin. This suggests that there are at least 3 species in the system.

**Figure S15**.

^1^H NMR temporal evolution studies with wool fabric (mSJ) in the solution. For comparison solution without wool is also reported. Here, aliquots are taken every 10 minutes from the dye bath for both cases, and subsequently quenched using sodium thiosulfate to suppress polymerization.

**Figure S16**.

Change in L* with different dye initialization time. Dye initiation time is the timepoint at which the fabric is added to the dye bath to interact with the PDA polymerizing solution. For instance 5 min refers to the fabric being added after 5 minutes from initiating the PDA polymerization.

**Figure S17.**

Temporal change in N/C and weight gain (%) during PDA dyeing

**Figure S18.**

ss proton NMR of pristine wool and PDA dyed wool.

**Table S1.**

Atomic weight percentages of elements recorded of dyed fabrics from XPS spectroscopy

**Table S2.**

# PDA dyeing parameters and results reported in literature compared to our study

# **Table S3.**

# Species distribution of mW calculated from high resolution XPS spectra

# **Table S4.**

# Parameters of different fabric architectures

# **Table S5.**

# Pearson correlation matrix shows fabric thickness and gcm strongly effect the L^*^ value. Furthermore, the values confirm the collinearity between variables gcm and gsm with thickness, while yarn twist have the minimum effect on the L* value.

# **Table S6.**

# Impact of fabric structures (mSJ and mDK) dyed with PDA on different spectral regions; visible. (400 – 800 nm), SWNIR (800 – 1500 nm) and LWNIR (1500 – 2500 nm) a. %R_avg_ b. %T_avg_ and c. %A_avg_

**Table S7**.

Atomic weight percentages of elements recorded for PDAmSJ with time. SP_20 min refers to mSJ fabric treated with sodium periodate for 20 minutes without dopamine HCl in the dye bath.
